# Resveratrol prevents antibody-induced apoptotic death of retinal cells through upregulation of Sirt1 and Ku70

**DOI:** 10.1186/1756-0500-1-122

**Published:** 2008-12-01

**Authors:** Thimmappa S Anekonda, Grazyna Adamus

**Affiliations:** 1 Department of Ophthalmology, Casey Eye Institute, School of Medicine, Oregon Health & Science University, Portland, Oregon 97239, USA

## Abstract

**Background:**

To determine whether resveratrol, a natural plant-derived drug, has protective effects against antibody-induced apoptosis of retinal cells *in vitro *and to provide insights on the mechanism of resveratrol protection.

**Findings:**

E1A.NR3 retinal cells pretreated with 40 μM resveratrol were grown in the presence of anti-recoverin (Rec-1), anti-enolase (Enol-1) antibodies, and normal purified immunoglobulins. When the cells were exposed to resveratrol before treatment with Enol-1 or Rec-1 antibodies, 30–55% more cells survived compared to the resveratrol-untreated cells. Western blotting showed a reduction in proapoptotic protein Bax in the cytoplasm and mitochondria of resveratrol-treated cells. Resveratrol-pretreated cells also showed a significant decrease in intracellular calcium and an inhibition of caspase-3 activity as compared to the untreated cells. Sirt1 expression was greatly reduced in the cells grown in the presence of Rec-1 and Enol-1, but it increased about five times in the resveratrol-pretreated cells. Immunocytochemistry revealed that Sirt1 expression in the cytoplasm and nucleus was colocalized with Ku70 expression in resveratrol-treated cells, suggesting possible interaction with each other in the cell. The pattern of the Ku70 cellular localization also overlapped with the Bax cellular localization in treated and untreated cells.

**Conclusion:**

*In vitro *protection of retinal cells from apoptosis by resveratrol occurred through multiple early molecular events, such as reduction of intracellular calcium levels, down-regulation of Bax, up-regulation of Sirt1 and Ku70 activities, and inhibition of caspase-3 activity. These findings will help designing future *in vivo *and pre-clinical treatments for autoimmune retinopathies.

## Background

Patients with autoimmune retinopathies (AR), including cancer-associated retinopathy (CAR), suffer from retinal degeneration and progressively lose their vision. Currently available corticosteroid and immunomodulation therapies have limited roles in modifying the progression of AR or CAR [1]. Therefore, a safe and reliable treatment is urgently needed for these patients. Furthermore, age is the strongest risk factor for the incidence of retinal degeneration in adult Americans [2]. The prevalence of vision impairments and blindness increases after the age of 40 and is particularly rapid after age 75 [3]. We believe that designing an effective therapy for the treatment of autoimmune retinopathies involves both understanding the disease mechanism and utilizing anti-aging mechanisms in therapeutics.

CAR and AR are associated with circulating autoantibodies [4,5]. The most common autoantibodies found in association with vision loss are against recoverin and α-enolase [5]. In both cases, an increased intracellular calcium ([Ca^+2^]_i_) caused by antibody triggered the apoptotic pathway, and in patients, it can lead to degeneration of photoreceptors in the retina [6-9]. In this study, we evaluated the effect of resveratrol, a polyphenolic phytoalexin, on levels of [Ca^+2^]_i _and on protection of retinal cells from antibody-induced apoptotic death *in vitro*.

Resveratrol has strong anti-aging properties and has been shown to play a neuroprotective role in several neurological disorders [10-14] by protecting brain cells from death. Recent studies also directly link the beneficial effects of resveratrol to prevention of vision loss [15-18]. These studies strongly suggest that resveratrol could be useful for treating vision and neurological disorders associated with diverse pathologies. The protective effects of resveratrol on the retinal cells were examined in the *in vitro *study using undifferentiated, immortalized rat retinal E1A.NR3 cells, which express markers specific for photoreceptors, bipolar cells, ganglion cells, and retinal glial cells [19].

The molecular mechanism of resveratrol in cellular protection is not fully understood. Resveratrol acts by inducing the anti-aging protein Sirt1 in organisms ranging from yeasts to mammals [20,21]. Sirt1 exhibits anti-apoptotic properties by deacetylating Ku70 protein in HEK293T kidney cells [22]. Ku70, a DNA repair protein present in the nucleus in its native deacetylated form, sequesters Bax in the cytoplasm, and thereby performs a protective role in the cell [23]. In our recent study on antibody-induced apoptosis in retinal cells, the upregulated Bax translocated to mitochondria and triggered mitochondria-mediated caspase-3-mediated apoptosis and ultimately caused retinal cell death [6,9]. We hypothesize that resveratrol upregulates Sirt1 and Ku70 in retinal cells and suppresses Bax in the cytoplasm, therefore protecting cells from apoptotic death induced by anti-retinal antibody.

## Methods

### MTT assay

E1A.NR3 cells [24] were grown in a 96-well microplate at a density of 2 × 10^4^/well in 100 μl volume with 0–40 μM resveratrol for 16 hrs. 0.8 mg/ml of Rec-1 or Enol-1 were added to the culture for another 72 hrs. The cell viability was measured as described in a prior study [25].

### Intracellular calcium assay

[Ca^2+^]_i _was measured using the Fluo-4 NW Calcium Assay (Molecular Probes) as previously described [6]. Briefly, 2 × 10^4^/well E1A.NR3 cells were grown in 96-well plates overnight. Then 100 μl Fluo-4 NW dye was added for 30 min at 37°C. After adding 50 μl/well of antibody (0.8 mg/ml of Enol-1, Rec-1, normal IgG) or thapsigargin (2 μM) alone, or after 15 min pre-treatment with 40 μM resveratrol, measurements were made using an FLx800 Microplate Fluorescence Reader (Bio-Tek Instruments, Inc.).

### Cell fractionation

Cells (25 × 10^4^) were pre-treated with 0–40 μM resveratrol for 16 hrs, followed by incubation with 0.8 mg/ml of Rec-1 or Enol-1 for 2 hrs. For total protein extraction, cells were harvested, lysed, and centrifuged for 30 min at 15,000 g. Cytosolic and mitochondrial fractions of cells treated with 0.8 mg/ml antibody for 45 min were obtained as described in [6]. The protein content in each fraction was determined using a BCA Protein Assay (Pierce).

### Western blotting

The blots containing 50 μg total, cytosolic or mitochondrial protein extracts were blocked in of 5% nonfat milk in 0.1% Tween 20/PBS for 1 hr and then HRE-conjugated antibodies were used as follows: 1:500 for mouse anti-Bax (Santa Cruz Biotech), 1:500 for rabbit anti-Sirt1 (Santa Cruz Biotech), 1:500 for goat anti-Ku70 (Santa Cruz Biotech), 1:1000 for goat anti-α-tubulin (Santa Cruz Biotech) and rabbit anti-Cox-IV (Cell Signaling) antibodies. Blots were developed using LuminiGOLD ECL Western Blotting Detection Kit (SgnaGen Lab) and exposed to an X-Omat film (Kodak) until dark bands appeared. Blots were analyzed by densitometry using Kodak Digital Science 1D Image Analysis software. α-tubulin was used as a control for cytoplasmic and total proteins, and Cox-IV for mitochondrial proteins.

### Immunofluorescence cytochemistry

Retinal cells were grown on a slide (2 × 10^4^/well) overnight pretreated with 40 μM resveratrol for 16 hrs and then apoptosis was induced with 0.8 mg/ml Rec-1 or Enol-1 for 2 hrs. Next, cells were washed, fixed in 4% paraformaldehyde, and permeabilized with 0.3% Triton X-100. After 30 min blocking with 1% BSA in PBS, one of the following antibodies was added: mouse anti-Bax, 1:50; rabbit anti-Sirt1, 1:200; or goat anti-Ku70, 1:50 (Santa Cruz Biotech, Inc.) at 4°C overnight. Secondary antibodies conjugated to fluorochromes (Alexa Fluor 488, green and Alexa Fluor 594, red) (Invitrogen) were added for 1 hr. The images were photographed under a Leica DM5000B fluorescence microscope.

### Determination of caspase-3 activity

5 × 10^5^/well cells were grown overnight and 40 μM resveratrol was added 16 hrs before the induction of apoptosis with 0.8 mg/ml Rec-1 or Enol-1 for 8, 16, and 24 hr in triplicate. DMSO (0.5%) was used as a control. Following antibody treatments, cells were harvested, lysed, and centrifuged. A 50 μl supernatant was used for determining caspase-3 activity using an EnzChek caspase-3 fluorescent assay (Molecular Probes). Caspase-3 activity was determined from a standard curve and normalized to the total protein content in each sample.

### Statistical analysis

Results are expressed as mean ± SE. The treatment differences were assessed by one-way ANOVAs and Mann-Whitney *t*-tests. A *P *value less than 0.05 was considered significant.

## Results

Our previously published studies have established specific timelines for antibody-induced cell death, including the rise of [Ca^2+^]_i _(3–30 min), expression of Bax (15–120 min), and activation of caspase-3 (16–24 hrs) in E1A-NR3 retinal cells following incubation with antibody [5,6,9,24].

**Figure 1:**
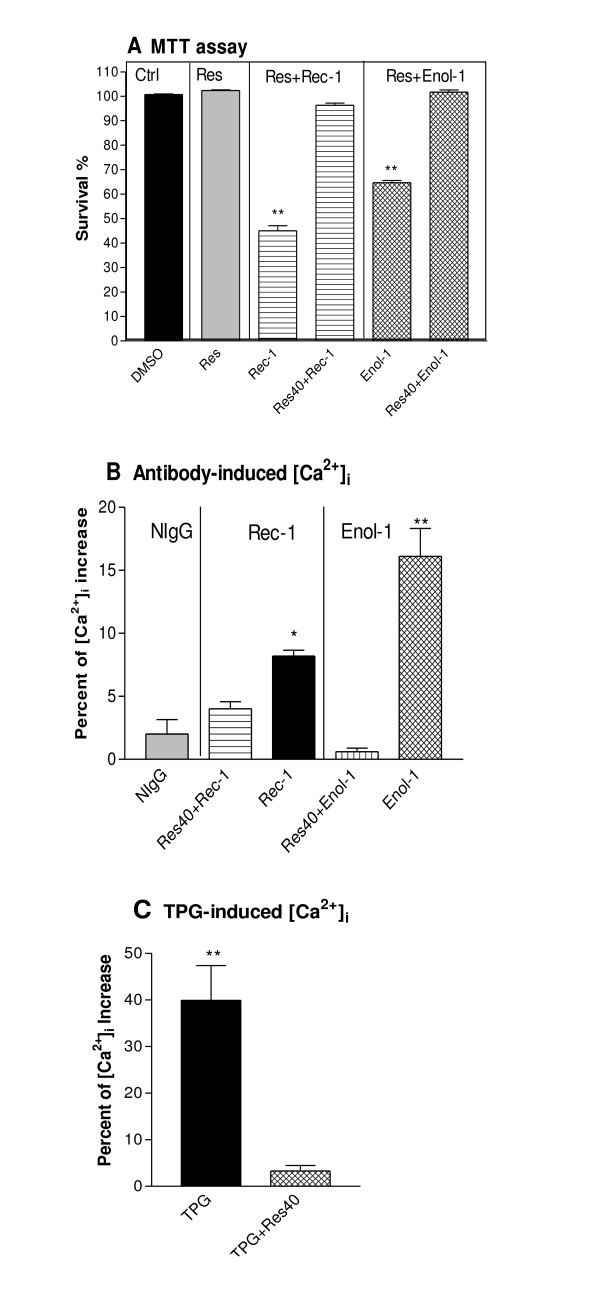
**Protection of retinal cells by resveratrol against antibody-induced cytotoxicity**. (A) E1A.NR3 cells were grown in 96-well plates (100 μl) for 72 hr in the presence of 0.8 mg/ml purified Rec-1 or Enol-1 alone, or antibodies plus 40 μM resveratrol (Res). Cell survival was measured by MTT assay. Each treatment was replicated three times, and the bars represent an average percentage of cell survival for 3 experiments. The statistical significance of each treatment was estimated against controls using one-way ANOVA and Mann-Whitney t-tests. ** = p < 0.01. (B) Effects of resveratrol on antibody-induced intracellular [Ca^2+^] levels. Cells were incubated in a fluorescent dye for 30 min before the addition of 50 μl of 0.8 mg/ml Rec-1 and Enol-1 or (C) 2 μM thapsigargin (TPG), individually or after 15 min pre-treatment with 40 μM resveratrol. Each treatment was replicated three times and the experiment was repeated thrice. The statistical significance of each treatment was estimated against DMSO controls using one-way ANOVA and Mann-Whitney t-tests. * = p ≤ 0.05; ** = p ≤ 0.01.

To determine whether resveratrol protects against Rec-1 or Enol-1 induced cytotoxicity, retinal cells were grown in the presence or absence of resveratrol and then exposed to Eno-1 and Rec-1. Fig 1A shows marked protective effects of 40 μM resveratrol on the antibody-induced apoptosis in retinal cells. Without resveratrol, Rec-1 significantly reduced cell survival to 45% and Enol-1 reduced the survival to 65% of the DMSO control (p < 0.01). To determine whether resveratrol acts as calcium channel blocker and inhibits [Ca^2+^]_i_, we determined free [Ca^2+^]_i _in antibody/resveratrol treated cells. Treatment of retinal cells with Rec-1 and Enol-1 caused small, but significant increases in [Ca^2+^]_i _by 8% and 16%, respectively (p < 0.01), compared to normal IgG and untreated control (Fig 1B). When the cells were pretreated with 40 μM resveratrol for 15 min and then treated with Rec-1 and Enol-1, the [Ca^2+^]_i _level stayed at statistically similar to the control levels, suggesting that resveratrol inhibited [Ca^2+^]_i _increases. To test if resveratrol could restore the SERCA function, we used thapsigargin (TPG), a specific inhibitor of SERCA, in the presence and absence of resveratrol [6]. 2 μM TPG caused a significant increase of [Ca^2+^]_i _in the control, indicating calcium release from intracellular stores in the endoplasmic reticulum. In contrast, 40 μM resveratrol pre-treatment effectively decreased the [Ca^2+^]_i _(Fig 1C), suggesting that resveratrol may function as a calcium channel blocker in the retinal cells.

**Figure 2:**
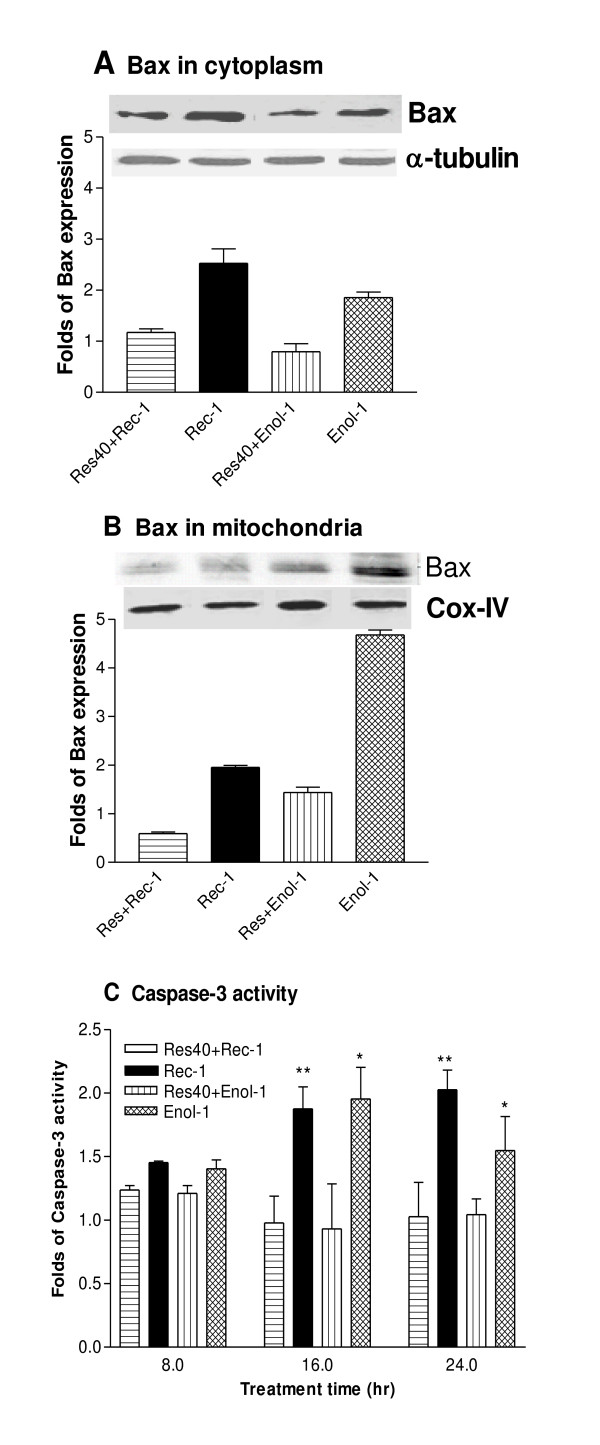
**Molecular effects of resveratrol on pro-apoptotic proteins in E1A.NR3 retinal cells**. Effects of resveratrol on Bax expression in the cytoplasm (A) and mitochondria (B). The retinal cells were treated with 40 μM resveratrol (Res) for 16 hrs followed by treatment with 0.8 mg/ml of Rec-1 and Enol-1 for 45 min. The protein expression was determined by Western blotting and the expression level is presented as a fold increase calculated from the intensity of each band compared to the untreated control after adjusting for the loading control using densitometry analyses. The bars represent an average of three experiments. (C) Effects of resveratrol on caspase-3 activity. The EnzChek Caspase-3 fluorescent assay was used to determine the protease activity of caspase-3 in E1A.NR3 cells in the presence and absence of 40 μM resveratrol pretreatment (16 hrs) followed by induction of apoptosis using 0.8 mg/ml of Rec-1 or Enol-1 for 8, 16, and 24 hrs. Each treatment was replicated three times and the experiment was repeated twice. The statistical significance of treatments under each time of exposure was estimated against vehicle control using two-way ANOVA and Mann-Whitney t-tests. * = p ≤ 0.05; ** = p ≤ 0.01.

To determine if resveratrol affects the expression of Bax, we analyzed levels of Bax in the presence or absence of 40 μM resveratrol followed by induction of apoptosis for 45 min. Fig 2A shows that in the cytoplasm of resveratrol-treated cells, Bax expression stayed at 1-fold compared to 2.5-fold increase in Rec-1 and 2-fold increase in Enol-1 treatments (Fig. 2A). Bax expression was 2- and 4.5-fold greater in the Rec-1 and Enol-1 treated mitochondria, respectively, as compared to untreated cells or cells pretreated with resveratrol prior to antibody treatment (Fig 2B). These results suggest that resveratrol may inihibit the overexpression of Bax in both cytoplasm and mitochondria as early as 45 min, and thereby preventing the mitochondria-mediated apoptosis. In the next experiment, we determined whether resveratrol affected caspase-3 activation in the retinal cells during apoptosis. Fig 2C shows caspase-3 activities in the cells exposed to 16-hr resveratrol treatment, and then exposed to Enol-1 or Rec-1 for 8, 16 and 24 hrs. There was no difference in the caspase-3 activity among the treatments after 8-hr; however, there was a significant reduction in caspase-3 activity in resveratrol-pretreated cells for 16- and 24-hr as compared to cells treated with Rec-1 (p < 0.01) and Enol-1 (p < 0.05) alone. The inhibition of caspase-3 activity was probably due to inactivation of Bax in the cytoplasm by resveratrol in the early steps of the apoptotic events.

**Figure 3:**
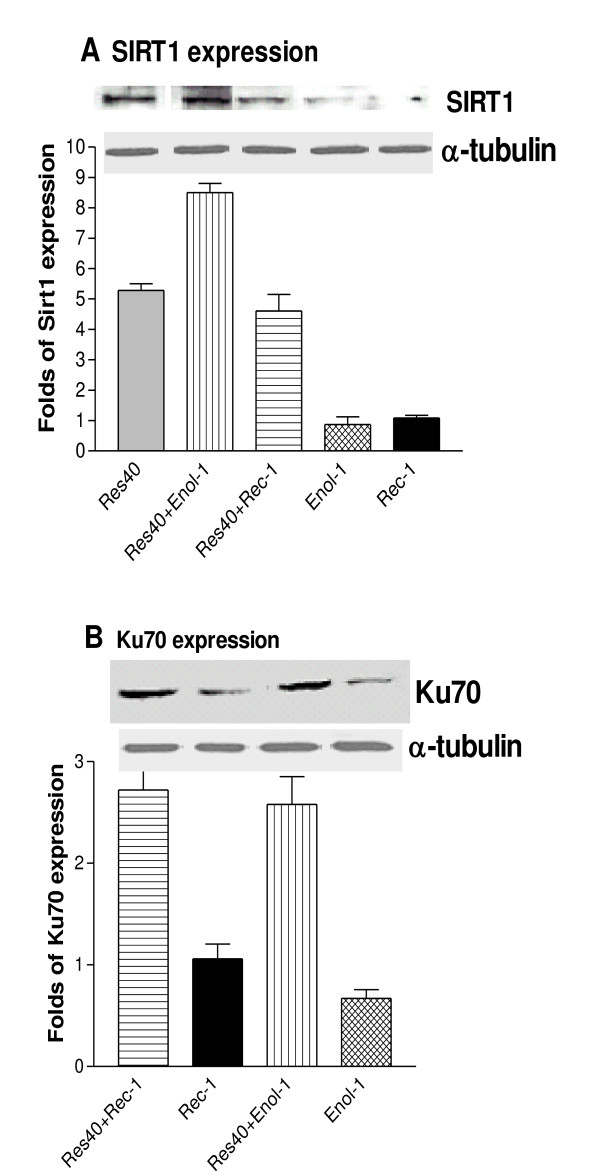
**Molecular effects of resveratrol on anti-apoptotic proteins**. Effects of resveratrol on (A) Sirt1 and (B) Ku70 expression in antibody-treated E1A-NR3 cells. The cells were pretreated with 40 μM resveratrol for 16 hrs followed by 2 hrs incubation in Enol-1 or Rec-1 antibodies. The protein cell extracts were analyzed by Western blotting using anti-Sirt1 and anti-Ku70 antibodies. Expression level is presented as a fold increase calculated from the intensity of each band compared to the untreated controls adjusted for the loading control using a densitometry analyses. The bars represent an average of three experiments.

Next, we examined the effects of resveratrol on Sirt1 and Ku70 expression during antibody-induced apoptosis. Fig 3A shows that about 5-fold Sirt1 increase in 40 μM resveratrol treated cells compared to untreated control, suggesting that resveratrol may function as an effective Sirt1 activator in retinal cells. About 4 to 8 times Sirt1 increase was observed in the resveratrol-pretreated cells that were subsequently exposed to Enol-1 or Rec-1. Sirt1 expression was at the control level in the Enol-1 and Rec-1 treatments. Ku70 expression was nearly 3 times greater in the resveratrol-pretreatment than in antibody alone (Fig 3B). These results show that both antibodies suppressed the expression of anti-apoptotic Sirt1 and Ku70 and resveratrol causes their upregulation, which lead to protection of the cells from apoptosis.

**Figure 4:**
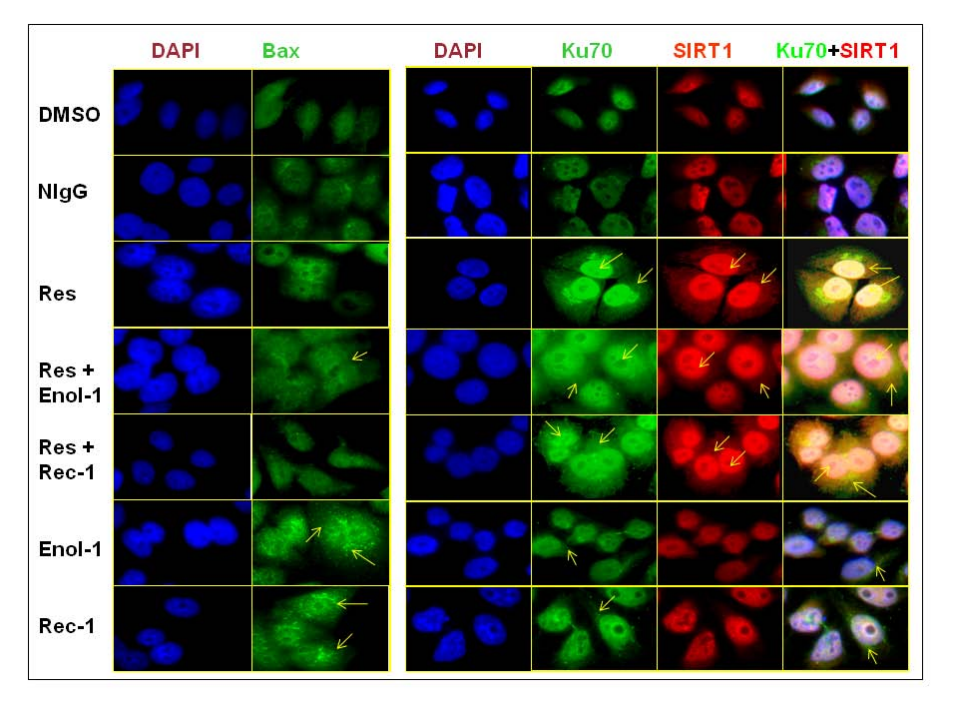
**Immunofluorescent analysis of pro-and anti-apoptotic proteins**. Immunofluorescent analysis of the expression of Bax (green), Ku70 (green), and Sirt1 (red) proteins in E1A.NR3 retinal cells in the presence and absence of 40 μM resveratrol pretreatment (16 hrs) followed by induction of apoptosis using 0.8 mg/ml of Rec-1 or Enol-1 for 2 hrs. The cells were incubated with anti-Bax, anti-Ku70 and anti-Sirt1 antibodies followed by incubation in appropriate fluorescent secondary antibodies. Nuclei were stained with DAPI (blue) for 10 min. The images were photographed under a Leica DM5000B fluorescence microscope. Each treatment was replicated three times and the experiment was repeated twice. The arrows indicate the expression of corresponding proteins in the cytoplasm of nucleus.

Fig 4 shows fluorescent images of nuclei stained with DAPI and immunofluorescent images of Bax, Ku70, and SIRT1 in cells treated with resverarol and antibody or antibody alone. DMSO or NIgG controls showed only a minimal expression of Bax in the cytoplasm and Ku70 and SIRT1 in the nucleus, but not in the cytoplasm. As expected, Bax was dramatically upregulated by the Rec-1 and Enol-1 and was seen as thick green patches both in the cytoplasm and in the peri nuclear regions, suggesting the presence of Bax also in the mitochondria. Both Ku70 and SIRT1 increased in the resveratrol-treated cells, whether or not the cells were grown with antibodies. There was a strong immunofluorescent staining of Ku70 and SIRT1 in the nucleus and cytoplasm, indicating their co-localization as shown by yellow color (Fig 4). Only a negligible Ku70 staining was observed in the cytoplasm of the cells treated with Enol-1 and Rec-1.

## Discussion and conclusion

**Figure 5:**
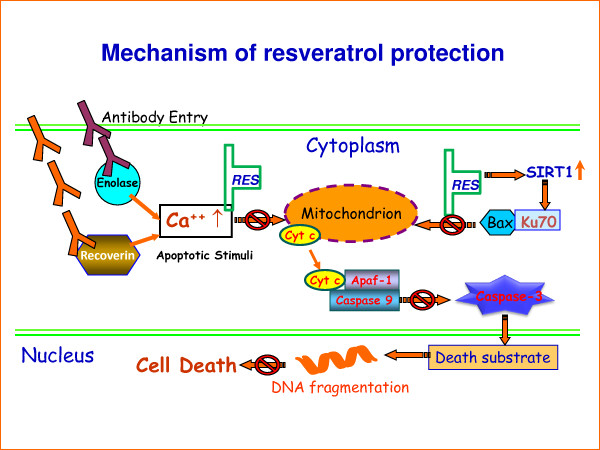
**A proposed molecular mechanism of resveratrol protection against antibody-induced apoptosis in retinal cells**. Antibodies enter the cell through endocytosis and induce an increase in intracellular calcium levels, which in turn trigger the mitochondria-mediated increase in caspase 3 activity and apoptotic death of retinal cells. Resveratrol blocked the intracellular calcium level and blocked the entry of Bax from cytoplasm to mitochondria, which subsequently short-circuited the caspase-3 activation and the cell from death. This figure has been modified from a previously published figure in [9].

In this study, we showed for the first time the effects of the anti-aging drug resveratrol against Rec-1 and Enol-1-induced retinal cell death, using an *in vitro *model of antibody-mediated retinopathy. Our findings demonstrate that by blocking the elevation of [Ca^2+^]_i _levels early in the apoptotic pathway, resveratrol reduced the expression of pro-apoptotic Bax in the cytoplasm and mitochondrion. This could be related to the increased expression of Sirt1 and Ku70 in resveratrol-treated cells, suggesting that the upregulation of these proteins interfered with the pro-apoptotic Bax movement to the mitochondria – essential steps for mitochondria-mediated apoptotic cell death [9,26,27]. Our findings support the hypothesis that Sirt1 deacetylates Ku70 and promotes its interaction with Bax in the cytoplasm, thus preventing Bax translocation to mitochondria [22]. Based on our results, we propose a mechanism of the resveratrol protection from antibody-induced apoptosis as illustrated in Fig 5, which shows that antibodies enter the cell by endocytosis and induce increased intracellular calcium levels, which in turn trigger the mitochondria-mediated apoptotic cell death [6,9,27]. Resveratrol blocks the influx of intracellular calcium and the entry of Bax from the cytoplasm to mitochondria, which subsequently prevents caspase-3 activation and the cell death. If this mechanism of the resveratrol protection is confirmed in animal models of retinopathy, resveratrol could be considered as a therapeutic drug for diseases, in which autoantibodies cause retinal cell death. These findings and future in vivo studies will serve as crucial pre-clinical experiments, upon which, clinical studies can be designed.

## Abbreviations

AR: Autoimmune retinopathy; CAR: Cancer-associated retinopathy; DMEM: Dulbecco's modified Eagle's medium; Enol-1: Anti-α-enolase antibody; FBS: Fetal Bovine Serum; MAb: Monoclonal antibody; PMCA: Plasma membrane ATPase Ca^2+ ^channel; Rec-1: Anti-recoverin antibody; RES: Resveratrol; RPE: Retinal pigment epithelium; SERCA: Sarco-Endoplasmic Reticulum Ca^2+^-ATPase channel; TPG: Thapsigargin

## Competing interests

The authors declare that they have no competing interests.

## Authors' contributions

TSA, conceived and designed the study, performed literature search, conducted experiments, analyzed and interpreted data, wrote and critically revised the manuscript. GA, participated in all stages of study design, data interpretations and critical revisions of the manuscript. All authors have read and approved the final manuscript.
